# PIBLUP: High-Performance Software for Large-Scale Genetic Evaluation of Animals and Plants

**DOI:** 10.3389/fgene.2018.00226

**Published:** 2018-08-14

**Authors:** Huimin Kang, Chao Ning, Lei Zhou, Shengli Zhang, Ning Yang, Jian-Feng Liu

**Affiliations:** ^1^National Engineering Laboratory for Animal Breeding, China Agricultural University, Beijing, China; ^2^Key Laboratory of Animal Genetics, Breeding and Reproduction, Ministry of Agriculture, China Agricultural University, Beijing, China; ^3^Department of Animal Genetics, Breeding & Reproduction, College of Animal Science and Technology, China Agricultural University, Beijing, China

**Keywords:** genetic evaluation, Message Passing Interface, Intel Math Kernel Library, preconditioned conjugate gradient, iteration on data, large-scale, multicore/multiprocessor

## Abstract

Today, the rapid increase in phenotypic and genotypic information is leading to larger mixed model equations (MMEs) and rendering genetic evaluation more time-consuming. It has been demonstrated that a preconditioned conjugate gradient (PCG) algorithm via an iteration on data (IOD) technique is the most efficient method of solving MME at a low computing cost. Commonly used software applications implementing PCG by IOD merely employ functions from the Intel Math Kernel Library (MKL) to accelerate numerical computations and have not taken full advantage of the multicores or multiprocessors of computer systems to reduce the execution time. Making the most of multicore/multiprocessor systems, we propose PIBLUP, a parallel, shared memory implementation of PCG by IOD to minimize the execution time of genetic evaluation. In addition to functions in MKL, PIBLUP uses Message Passing Interface (MPI) shared memory programming to parallelize code in the entire workflow where possible. Results from the analysis of the two datasets show that the execution time was reduced by more than 80% when solving MME using PIBLUP with 16 processes in parallel, compared to a serial program using a single process. PIBLUP is a high-performance tool for users to efficiently perform genetic evaluation. PIBLUP with its user manual is available at https://github.com/huiminkang/PIBLUP.

## Introduction

Genetic evaluation is widely accepted as an efficient method to improve genetically complex traits and is increasingly applied to the breeding programs of animals and plants. Solving mixed model equations (MMEs) is a major part of genetic evaluation. Statistical models used in genetic evaluations are usually complicated, which include either multiple correlated effects within the same trait or multiple traits analyzed simultaneously. An example of the former is random regressions nested within an additive genetic effect or within a permanent environmental effect in test-day models for milk production traits (Schaeffer et al., 2000). Examples of the latter are multiple-trait analyses of type traits (Tsuruta et al., 2011), fertility traits (Liu et al., 2008), and a multiple-trait multiple-lactation test-day model for milk production traits (Schaeffer et al., 2000). Moreover, with genomic selection, the relationship matrix based on markers becomes denser than that based on pedigree. All the aspects mentioned above make solving MMEs a time-consuming task. In animal breeding, large MMEs are usually solved using a preconditioned conjugate gradient (PCG) by iteration on data (IOD). PCG is easy to implement and has a superior convergence rate compared to other commonly used iterative methods, such as the Gauss–Seidel method (Tsuruta et al., 2001) and the Gauss–Seidel second-order Jacobi method (Strandén and Lidauer, 1999). Incorporating the IOD technique into the PCG algorithm avoids the high memory consumption of storing a large coefficient matrix of MME and enables the implementation of large-scale genetic evaluation (Strandén and Lidauer, 1999).

With the accumulation of phenotypic and genomic data, current genetic evaluations are more time consuming than those that existed before the genomic era, especially in situations where the aforementioned complicated models are employed. Parallel computing is a practically workable solution to this problem in which a computational task is typically divided into several similar, independent subtasks whose results are combined afterwards.

The Intel Math Kernel Library (MKL) (https://software.intel.com/en-us/intel-mkl) provides optimized and threaded math functions to obtain immediate performance benefits. Among the currently available software packages, as far as we know, BLUPF90 (Misztal et al., 2002) and DMU (Madsen and Jensen, 2013) employed MKL to speed up computations.

When the entire MME system is stored in main memory, almost all the steps of the PCG algorithm can be parallelized using math functions in the MKL library. However, with the implementation of an IOD technique, one of the most time-consuming parts can no longer be parallelized with MKL. In this case, Message Passing Interface (MPI) (MPI Forum, 2012) provides a good way to implement parallel programming with the computational load manually partitioned across multiple processes.

Taking full advantage of multicore/multiprocessor systems, we present PIBLUP, a C implementation of PCG by IOD using both MPI parallel programming and MKL math functions in order to reduce the execution time of genetic evaluations and facilitate large-scale analyses. PIBLUP with its user manual is available at https://github.com/huiminkang/PIBLUP.

## Methods

PIBLUP is written in C and is supported on a Linux operating system with a shared memory architecture. PIBLUP is driven by a parameter file, where the MME information is specified in sections defined by keywords (see examples in user manual at https://github.com/huiminkang/PIBLUP).

**Figure 1:**
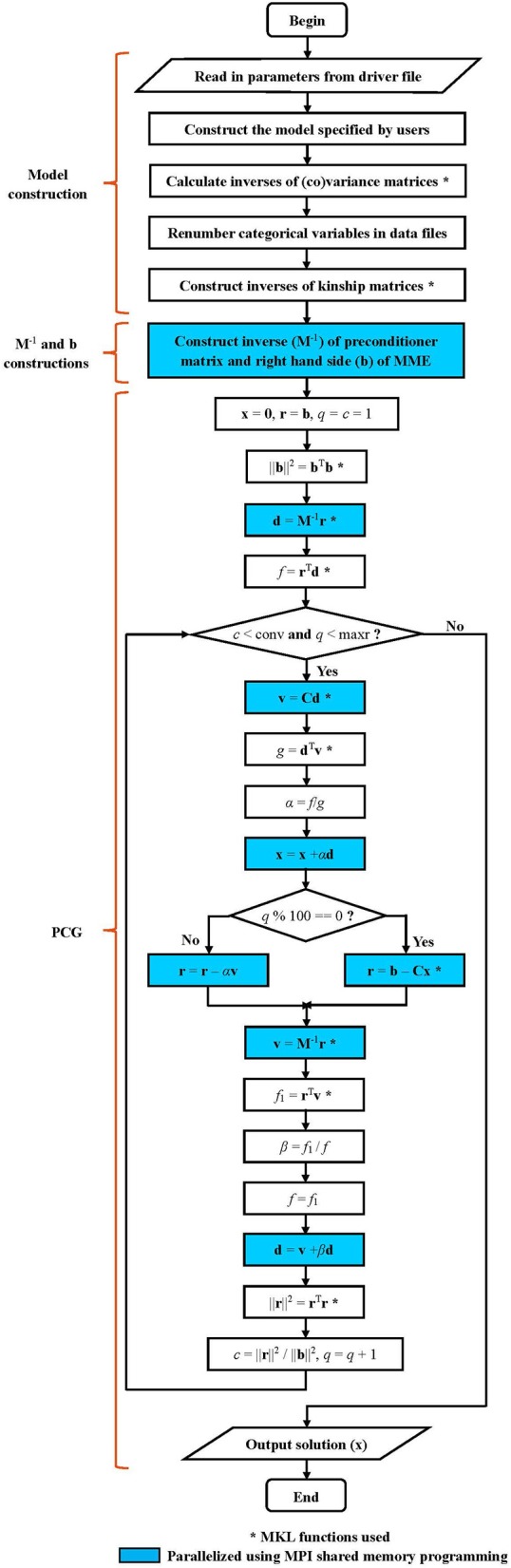
Workflow diagram for PIBLUP. The mixed model equation to be solved is **Cx** = **b**. The preconditioner matrix is **M**, a block diagonal matrix formed from the coefficient matrix **C**. Implementation of steps marked by “*” employs math functions in Intel Math Kernel Library. Code in parts of **M**^−1^ and **b** constructions and PCG are parallelized using Message Passing Interface shared memory programming. Steps in cyan color are executed by all the processes to reduce runtime, and those in white are executed by the master process. All the processes execute the two conditional statements in diamonds.

The parallel version of PIBLUP is programmed based on an optimized serial version. The workflows of these two versions (shown in Figure 1) are the same, except that MPI parallel programming was not used in the serial version. Details of the workflow are explained as follows.

### Statistical model construction

Based on the specified parameter file, PIBLUP constructs the statistical model used in genetic evaluation. Then, (co)variance matrices for random effects are inverted to facilitate the following computations. To be more user-friendly, PIBLUP renumbers categorical variables in data files, such as phenotype file, pedigree, and genotype file, permitting the use of raw data including alpha-numeric characters in input files.

Subsequently, inverses of kinship matrices are constructed, including the inverse of a numerator relationship matrix (**A**) based on pedigree (Henderson, 1975, 1976), the inverse of a genomic relationship matrix (**G**) based on genomic information (VanRaden, 2008) and the inverse of a hybrid relationship matrix (**H**) of **A** and **G** (Legarra et al., 2009; Christensen and Lund, 2010). In addition to these commonly used matrices, PIBLUP can leverage user-specified kinship matrices (their inverse matrices stored in input files) alternatively.

In PIBLUP, the **G** matrix is constructed utilizing the first method of VanRaden (2008) as follows:

(1) $$G=\frac{(S-P)(S-P{)}^{\prime }}{2\sum_{j=1}^{n}p_{j}(1-p_{j})},$$

where **S** is a matrix of SNP genotypes for each individual, **P** is a matrix of two times the observed allele frequency of the second allele *p* at locus *j* (*p*_*j*_), and *n* is the number of SNPs used in the construction of **G**. In PIBLUP, the **G** matrix is explicitly inverted. PIBLUP has two options to ensure that **G** is invertible. First, a small arbitrary value (specified in the parameter file, e.g., 0.01) can be added to the diagonals of **G**. Moreover, the nearest positive definite matrix of **G** could be computed using Higham's method (Higham, 2002) (specified in the parameter file).

The inverse of the **H** matrix in PIBLUP is constructed as follows (Legarra et al., 2009; Christensen and Lund, 2010):

(2) $$H^{-1}=A^{-1}+\left[\begin{array}{cc} 0 & 0\\ 0 & \tau {\left[\left(1-w\right)G^{*}+wA_{22}\right]}^{-1}-\omega A_{22}^{-1} \end{array}\right],$$

where G^*^ = β**G**+α with α and β are calculated from the system of equations:

(3) $$\{\begin{array}{c} \text{Avg}\left(\text{diag}\left(G\right)\right)\beta +\alpha =\text{Avg}\left(\text{diag}\left(A_{22}\right)\right)\\ \text{Avg}\left(\text{offdiag}\left(G\right)\right)\beta +\alpha =\text{Avg}\left(\text{offdiag}\left(A_{22}\right)\right) \end{array},$$

where Avg(diag(**B**)) and Avg(offdiag(**B**)) represent the average values of diagonal and non-diagonal elements of matrix **B**, respectively (Christensen et al., 2012). Matrix **A**_22_ is the partition of **A** and subscript 2 represents genotyped individuals. The adjustment of **G** is to avoid the potential incompatibility in scale between **G** and **A**_22_. Three parameters τ, *w*, and ω, whose values are specified by users, are set to achieve better accuracy and lower bias by fine-tuning (Tsuruta et al., 2011; Harris et al., 2012).

### Preconditioner construction in parallel

Let the MME be:

(4) $$\left[\begin{array}{cc} X^{\prime }R^{-1}X & X^{\prime }R^{-1}Z\\ Z^{\prime }R^{-1}X & Z^{\prime }R^{-1}Z+G^{-1}⊗G_{0}^{-1} \end{array}\right]\left[\begin{array}{c} \hat{f}\\ \hat{u} \end{array}\;\right]=\left[\begin{array}{c} X^{\prime }R^{-1}y\\ Z^{\prime }R^{-1}y \end{array}\right].$$

The coefficient matrix can be partitioned into two parts:

(5) $$\begin{array}{l} \left[\begin{array}{cc} X^{\prime }R^{-1}X & X^{\prime }R^{-1}Z\\ Z^{\prime }R^{-1}X & Z^{\prime }R^{-1}Z+G^{-1}⊗G_{0}^{-1} \end{array}\right]\\ =\left[\begin{array}{cc} X^{\prime }R^{-1}X & X^{\prime }R^{-1}Z\\ Z^{\prime }R^{-1}X & Z^{\prime }R^{-1}Z \end{array}\right]+\left[\begin{array}{cc} 0 & 0\\ 0 & G^{-1}⊗G_{0}^{-1} \end{array}\right], \end{array}$$

where the first part, the least square part, is constructed based on observations and the second part is based on the inverse of kinship matrix **G**^−1^ and the inverse of the (co)variance matrix for the random effect ${\text{G}}_{0}^{-1}$. **X** and **Z** are the design matrices of fixed effects **f** and random effects **u** respectively, **R** is the residual (co)variance matrix, and ⊗ is the Kronecker product. If the random effect is not a genetic effect, **G**^−1^equals the identity matrix **I**.

A block diagonal preconditioner **M** based on the coefficient matrix is used in PIBLUP to improve the convergence rate of PCG. The blocks of **M** are due to traits, resulting in dense blocks of the *t*×*t* matrix, where *t* is the number of traits. Both **M** and **M**^−1^ are constructed using MPI shared memory programming.

During the construction of **M**, PIBLUP stores the upper triangles of blocks in memory. A region of shared memory is allocated for **M** by a master process. All processes can access it without communication via a network. According to the partition of the coefficient matrix, the construction of **M** involves two tasks. All processes participate in both tasks. In the first task, the translated values are added to their corresponding positions based on each phenotypic record. All the processes read the phenotype file simultaneously, but each process calculates the addend and performs addition operations for specific positions. PIBLUP assigns at least one position to each process and tries to ensure an even distribution of the number of addition operations. The second task is adding values to **M** for each level of random effects. PIBLUP distributes work among processes according to the total number of levels and the number of processes.

After **M** has been constructed, its inverse is computed by inverting each diagonal block it contains. This is also implemented by all of the processes in parallel, with each process computing inverses for nearly an equal number of blocks. The inverse is calculated using the MKL math functions LAPACKE_dgetrf and LAPACKE_dgetri. Finally, **M**^−1^ is stored in disk.

While phenotypic records are processed in the construction of **M**, all processes construct the right-hand side (**b**) of MME in a similar manner in parallel. Specifically, **b** is stored in the region of shared memory allocated by the master process. All processes participate in adding translated values to their corresponding positions in **b** based on phenotypic records. Processes handle the same positions that were assigned in the construction of the least square part of **M**.

### Implementation of PCG by IOD in parallel

As shown in Figure 1, PIBLUP begins to solve MME using PCG by IOD after **M**^−1^ and **b** have been constructed. The parallelization of this process is realized using MPI shared memory programming and MKL functions (indicated by “^*^” in Figure 1). All the processes execute the steps in blue in Figure 1 in parallel to accelerate the computational speed.

With an IOD technique (Strandén and Lidauer, 1999), the coefficient matrix of MME (**C**) does not require to be stored in memory. Therefore, the memory consumption is dominated by five vectors, i.e., right-hand side vector **b**, residual vector **r**, search direction vector **d**, solution vector **x**, and vector **v**. With MPI shared memory model, these five vectors are stored in the shared memory of the master process and can be accessed by all processes. Each process only need to allocate an area of shared memory to store the intermediate vector **i**, which is used in the calculations of **Cd** and **Cx**.

The master process computes the inner products, i.e., **b**^*T*^**b**, **r**^*T*^**d**, **d**^*T*^**v**, **r**^*T*^**v**, and **r**^*T*^**r**, using the MKL function cblas_ddot. All the processes participate in the computation of **M**^−1^**r**, using the MKL function cblas_dsymv. As **M**^−1^ is a block diagonal matrix, each process performs an equal number of multiplications of blocks and vectors. **M**^−1^is read from disk each time.

The heaviest computation step in PCG is the multiplication of the coefficient matrix (**C**) and vector **d** or **x**. As shown in Equation [5], the **C** matrix can be partitioned into two parts. After being read from disk, the *j*th phenotypic record is translated into **x**_*j*_ and **z**_*j*_. Therefore,

(6) $$\begin{array}{l} \text{​​​​​​}Cd\text{​}=\left[\begin{array}{cc} X^{\prime }R^{-1}X & X^{\prime }R^{-1}Z\\ Z^{\prime }R^{-1}X & Z^{\prime }R^{-1}Z \end{array}\right]d+\left[\begin{array}{cc} 0 & 0\\ 0 & G^{-1}⊗G_{0}^{-1} \end{array}\right]d\text{​​​​​​​}\\ \;=\sum_{j=1}^{q}w_{j}R_{j}^{-1}{w^{\prime }}_{j}d+\left[\text{​}\begin{array}{cc} 0 & 0\\ 0 & \left(G^{-1}⊗I_{G_{0}^{-1}}\right)\left(I_{G^{-1}}⊗G_{0}^{-1}\right)d \end{array}\text{​}\right], \end{array}$$

where ${w^{\prime }}_{j}=\left[\begin{array}{cc} {x^{\prime }}_{j} & {z^{\prime }}_{j} \end{array}\right]$, *q* is the number of records, and ${\text{I}}_{{\text{G}}_{0}^{-1}}$ and ${\text{I}}_{{\text{G}}^{-1}}$ are identity matrices of orders equal the orders of ${\text{G}}_{0}^{-1}$ and **G**^−1^, respectively. The products $t_{j}=w_{j}R_{j}^{-1}{w^{\prime }}_{j}d$ and $\text{s}=\left({\text{G}}^{-1}⊗{\text{I}}_{{\text{G}}_{0}^{-1}}\right)\left({\text{I}}_{{\text{G}}^{-1}}⊗{\text{G}}_{0}^{-1}\right)\text{d}$ are both calculated from the right to the left (Strandén and Lidauer, 1999), i.e.,

(7) $$t_{j1}={w^{\prime }}_{j}d;\;t_{j2}=R_{j}^{-1}t_{j1};\;t_{j}=w_{j}t_{j2},$$

and

(8) $$\begin{array}{r} {\text{s}}_{1}=\left({\text{I}}_{{\text{G}}^{-1}}⊗{\text{G}}_{0}^{-1}\right)\text{d};\;\text{s}=\left({\text{G}}^{-1}⊗{\text{I}}_{{\text{G}}_{0}^{-1}}\right){\text{s}}_{1}. \end{array}$$

As phenotypic records are independent of each other, all processes read nearly equal numbers of records and calculate the corresponding **t**_*j*_. Similarly, the computations of **s**_1_ and **s** are assigned to processes according to the number of levels of random effects and the number of non-zero elements in **G**^−1^, respectively.

In each process, the values of vectors **t**_*j*_ and **s**_1_ are stored in the intermediate vector **i**. Then, vector **i** for **t**_*j*_ is summed up across processes and stored in vector **v** in the master process. The value of **i** for **s** from each process is directly added to **v** in the master process, as different processes cannot have concurrent addition operations on the same position in **v**.

### Convergence criterion

The convergence criterion used in PIBLUP is the relative average difference between the right-hand side and left-hand side as in the study of Tsuruta et al. (2001):

(9) $$\begin{array}{r} c=\frac{{\left||\text{b}-\text{C}\text{x}|\right|}^{2}}{{\left||\text{b}|\right|}^{2}}, \end{array}$$

where ||**y**|| is the length of vector **y** and ${\left||\text{y}|\right|}^{2}=\underset{i}{\sum }y_{i}^{2}$. The **b**–**Cx** in the numerator is approximated by **r**^*k*^ = **r**^k−1^ – α**v**, where *k* means the *k*th round. The residual vector **r** is updated using the exact formula **r** = **b** – **Cx** every 100 rounds to eliminate accumulated rounding errors.

### Application

We evaluated the performance of PIBLUP using two datasets of different sizes, and two commonly used statistical models in genomic selection were employed.

In the first dataset, there were 7,556 individuals with 72,507 markers genotyped. A total of 5,334 individuals among them have de-regressed proofs (DRPs) and corresponding weights (Garrick et al., 2009) for at most three traits. We used multi-trait genomic best linear unbiased prediction (GBLUP) method (VanRaden, 2008) to predict genomic estimated breeding values (GEBV):

(10) $$y=\mu 1_{n}+Zg+e,$$

where **y** is a vector of DRP of three traits, μ is the overall mean, **1**_*n*_ is a vector of *n* ones, **g** is the vector of additive genomic effects with a distribution of *N*(**0**, **G**⊗**G**_0_), **Z** is the corresponding incidence matrix, and **e** is the vector of random residuals with a distribution of *N*(**0**, **D**⊗**R**). **G** is a genomic relationship matrix. **D** is a diagonal matrix with diagonal elements $\frac{1}{weight}$. **G**_0_ and **R** were the (co)variance matrices for additive genomic effects and residuals, respectively.

The second dataset contained test-day records of three milk production traits in the first three lactations of dairy cattle. There were 3,571,661 individuals in pedigree and 7,847,612 test-day records in the phenotype file. Genotypic data from the first dataset were included in this dataset. The statistical model used was the multiple-trait multiple-lactation single-step random regression model (Kang et al., 2016):

(11) $$\begin{array}{r} \text{y}={\text{X}}_{1}{\text{b}}_{1}+{\text{X}}_{2}{\text{b}}_{2}+\text{Q}\text{a}+\text{Z}\text{p}+\text{e}, \end{array}$$

where **y** is the vector of observations, **b**_1_ is the vector of the herd-test date-parity effect, **b**_2_ is the vector of fixed regressions for calving age-season-parity effect, **a** and **p** are vectors of random regressions for additive genetic effect and permanent environmental effect, **X**_1_, **X**_2_, **Q**, and **Z** are design matrices of **b**_1_, **b**_2_, **a**, and **p**, respectively; and **e** is the vector of residuals. We adopted Legendre polynomials of the third order for random regressions for permanent environmental effect and Legendre polynomials of the fourth order for fixed and additive genetic regressions. It was assumed that

(12) $$\mathrm{var}\left[\begin{array}{c} a\\ p\\ e \end{array}\right]=\left[\begin{array}{ccc} H⊗G_{0} & 0 & 0\\ 0 & I⊗P & 0\\ 0 & 0 & R \end{array}\right],$$

where **G**_0_ (45 × 45) and **P** (36 × 36) are (co)variance matrices of additive genetic and permanent environmental regression coefficients, and **H** is the aforementioned hybrid relationship matrix of **A** and **G**, with τ = 1.6, *w* = 0.1, and ω = 0.5. The lactation was divided into four periods, and residual (co)variance matrices **R** were assumed to be the same within each period.

Tests were performed on a Linux server, with a shared memory architecture and a total memory size of about 529 GB. It had 64-bit Intel Xeon E7-4820 processors, each with a base frequency of 2.00 GHz.

## Results and discussion

The primary motivation for developing PIBLUP lies in reducing the runtime of genetic evaluation by using several concurrent processes. We have compared the EBV or GEBV predicted using PIBLUP and DMU in analyses of all the datasets we obtained. The correlation between results of the two software packages was 1.00 in all the cases. Table 1 shows the runtime of the serial and parallel versions of PIBLUP in the analyses of the two datasets, for three different parts in Figure 1. For each part of PIBLUP, the program using four processes was faster than either program that run with one process, except during the construction of **M**^−1^ and **b** for dataset 1. This exception occurred owing to the different speeds of file management functions used in the serial version and the parallel version. The effect was significant when the total runtime was short in constructions of **M**^−1^ and **b** for dataset 1 (0.09 min). Although the part of the model construction in PIBLUP merely employed MKL math functions to accelerate the speed of the matrix inversion (Figure 1), the time saved was about 60 and 40% for datasets 1 and 2, respectively. This can be explained by the fact that the computational load in this part was mainly due to the inversion of **G** matrix, which is reflected by the time spent in the kinship matrix inversion step accounting for about 90 and 66% of the total runtime for datasets 1 and 2, respectively. Solving MME by PCG is the most time-consuming part, as many rounds of iterations are required to reach convergence in practical applications. With the convergence criterion of 10^−13^, the rounds of iterations required were 2,252 and 628 for datasets 1 and 2, respectively. For this part, the parallel version with four processes used 31 and 49% of the runtime of the serial program for datasets 1 and 2, respectively. One reason why the ideal percentage of 25% was not achieved was due to some existing overhead, such as synchronization. Moreover, for dataset 2, the number of equations in MME during the analysis was 185 million, which is significantly greater than that in dataset 1. Therefore, the effect of the aforementioned different file input/output speeds of the serial and parallel versions of PIBLUP was more significant in the analysis of dataset 2, as more data were read from disk.

**Table 1:** Runtime (min) of different parts (model construction, **M**^−1^ and **b** constructions, and PCG) in PIBLUP in analyses of datasets 1 and 2.

**Program^a^**	**Dataset 1**^b^			**Dataset 2**^c^		
	**Model construction**	**M^−1^ and b constructions**	**PCG^d^**	**Model construction**	**M^−1^ and b constructions**	**PCG^d^**
Serial	21.97	0.09	0.61	36.56	14.45	87.21
Parallel (1)	21.97	1.56	0.62	36.56	17.36	98.76
Parallel (4)	8.63	0.41	0.19	22.57	5.47	42.61

a *Serial version (Serial), parallel version of PIBLUP with a single process (Parallel (1)) and four processes (Parallel (4)) were tested*.

b *There were 22,671 equations in mixed model equations for analysis of dataset 1*.

c *There were 185,048,637 equations in mixed model equations for analysis of dataset 2*.

d *PCG was iterated 50 rounds for easy comparison*.

In genetic and genomic evaluations, BLUPF90 (Misztal et al., 2002) and DMU (Madsen and Jensen, 2013) are the two most commonly used software packages. As the blupf90 program from BLUPF90 package and DMU4 program from DMU package are well developed and freely available, we compared the total runtime of PIBLUP (parallel version) with these two programs in the analysis of dataset 1. Table 2 shows the total runtime of different software packages run with a single process and four processes. With the maximum number of processes set to one, the runtime of PIBLUP, blupf90 and DMU4 was about 51, 85, and 58 min, respectively. According to the user manual of BLUPF90, PCG is the default solver in blupf90 and the fastest one. We chose PCG as the solver when blupf90 was employed. For dense MME, as those in the analysis of dataset 1, DMU4 uses LAPACK subroutines to reach a solution. Therefore, the different runtime of the three software packages may result from the different solving methods they employ. Moreover, as previously mentioned, it is time consuming to construct the inverse of **G** matrix in the analysis of dataset 1. DMU4 saved a larger amount of time because it read in a file containing the inverse of **G** matrix instead of constructing it on its own. In contrast, PIBLUP and blupf90 computed the inverse of **G** matrix based on the genotypes of SNPs. In addition, blupf90 consumed some time to calculate certain statistics of the G matrix and performed some quality control. Considering the factors mentioned above, the differences between the runtime of PIBLUP, blupf90 and DMU4 are acceptable. In contrast to PIBLUP, which uses the IOD technique, blupf90 and DMU4 set up equations in memory. Therefore, blupf90 and DMU4 are not suitable to solve very large MMEs, such as the MME in the analysis of dataset 2.

**Table 2:** Runtime (min) of PIBLUP, BLUPF90 and DMU in the analysis of dataset 1.

**No. of processes**	**PIBLUP^a^**	**BLUPF90^b^**	**DMU^c^**
1	51.49	84.97	58.18
4	17.61	49.87	38.79

a *Parallel version of PIBLUP*.

b *The blupf90 program from BLUPF90 package was employed*.

c *The DMU4 program from DMU package was employed*.

As presented in Table 2, runtime was reduced for all the three programs when the maximum number of processes increased from one to four. The execution time was reduced by approximately 66%, 41%, and 33% for PIBLUP, blupf90 and DMU4, respectively. To use multiple processes in parallel, PIBLUP employs both MPI parallel programming and MKL math functions (Figure 1), but blupf90 and DMU4 merely employ MKL math functions. With MPI, PIBLUP can further manually partition computational load across multiple processes (Figure 1). Therefore, PIBLUP saved a larger percentage of time compared to blupf90 and DMU4 when four processes were available. As aforementioned, not all steps in the workflow can be parallelized, and computing the inverse of **G** matrix is time consuming. Intel MKL provides parallelized math functions to compute the inverses of matrices. Program using these functions can reduce the runtime efficiently. As DMU4 doesn't construct the inverse of **G** matrix, the percentage of time saved for DMU4 with four processes was less than that for PIBLUP and blupf90.

**Figure 2:**
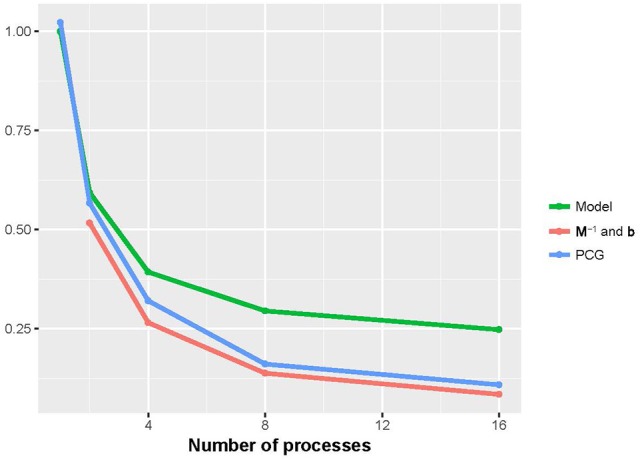
Relative runtime of each part (model construction, **M**^−1^ and **b** constructions, and PCG) in parallel version of PIBLUP against runtime of their serial counterparts by the number of processes in analyses of dataset 1. In **M**^−1^ and **b** constructions, relative time was calculated against the parallel version using a single process. Model, model construction; **M**^−1^ and **b**: **M**^−1^ and **b** constructions.

**Figure 3:**
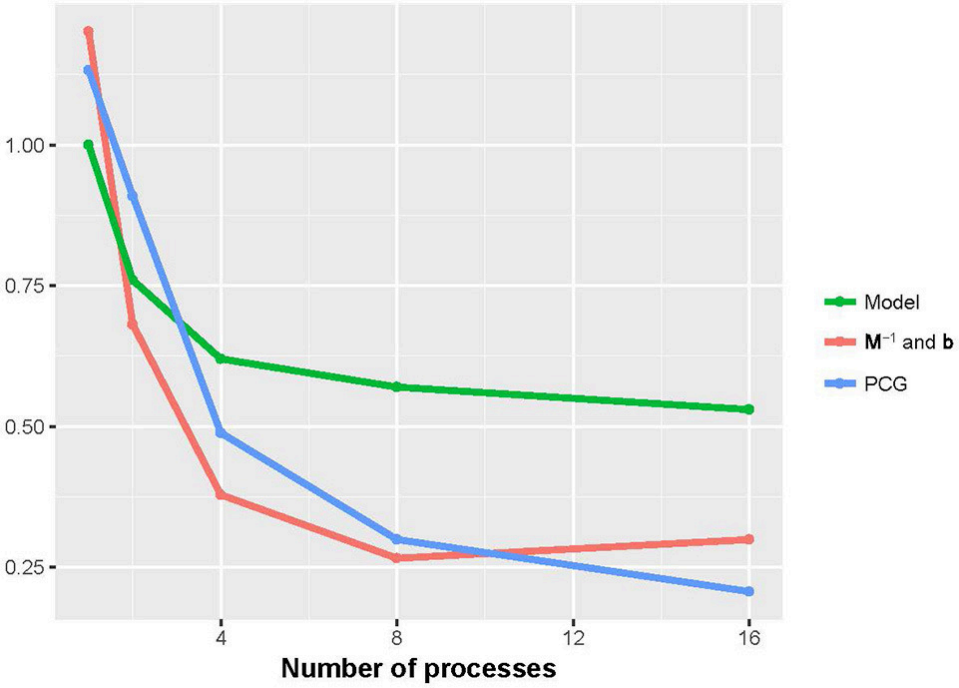
Relative runtime of each part (model construction, **M**^−1^ and **b** constructions, and PCG) in parallel version of PIBLUP against runtime of their serial counterparts by the number of processes in analyses of dataset 2. Model, model construction; **M**^−1^ and **b**: **M**^−1^ and **b** constructions.

Figures 2, 3 show that the runtime of each part of PIBLUP decreased as the number of processes increased. For the last two parts of PIBLUP, the execution time was reduced by more than 70% when 16 processes were used in parallel. As shown in Figure 1, not all the steps were parallelized, particularly those in the first step. As stated in Amdahl's law (Amdahl, 1967), the sequential steps limiting the runtime can be reduced. Therefore, the percent of runtime saved in the first part was smaller than that of the other two parts. When considering all the parts of PIBLUP, thread managements hindered the execution time from decreasing linearly with the number of processes.

As computer systems with a shared memory architecture are common and the number of cores currently continue to increase, PIBLUP chose to use MPI-3 shared memory programming to parallelize code. Implementations using MPI shared memory programming depend on a shared memory architecture and enable regions of shared memory allocated by one process to be accessed by other processes without communication across a network. Compared to point-to-point communication used in Stranden and Lidauer's study (Strandén and Lidauer, 2001), shared memory programming has the following benefits: (1) time for communication over a network among processes is saved; (2) processes can store less variables in local memory so that memory consumption is reduced; and (3) programming becomes relatively easy. For programs running in parallel and using shared memory programming, there is no clear method for calculating the actual total memory used by all processes. Therefore, we did not compare the memory usage herein. However, as processes other than the master process only need to store an intermediate vector **i** and other variables of small sizes, the expected total memory usage is relatively low.

## Author contributions

HK and J-FL developed software, performed statistical analysis, and wrote the manuscript. CN, LZ, SZ, and NY interpreted the results of analyses and edited the manuscript. J-FL conceived the study, supervised the work, and edited the manuscript.

### Conflict of interest statement

The authors declare that the research was conducted in the absence of any commercial or financial relationships that could be construed as a potential conflict of interest.
